# The Influence of Various Adhesive Systems and Polishing Methods on Enamel Surface Roughness after Debonding of Orthodontic Brackets: A Three-Dimensional In Vitro Evaluation

**DOI:** 10.3390/ma16145107

**Published:** 2023-07-20

**Authors:** Tereza Křivková, Antonín Tichý, Hana Tycová, Josef Kučera

**Affiliations:** 1 Institute of Dental Medicine, First Faculty of Medicine of the Charles University and General University Hospital in Prague, Kateřinská 32, 121 11 Prague, Czech Republic; tereza.krivkova@vfn.cz (T.K.); antonin.tichy@lf1.cuni.cz (A.T.); hana.tycova@vfn.cz (H.T.); 2 Department of Orthodontics, Faculty of Medicine and Dentistry, Palacký University Olomouc, Palackého 700/12, 779 00 Olomouc, Czech Republic

**Keywords:** orthodontics, adhesives, polishing, enamel surface roughness, CLSM, dentistry, dental materials

## Abstract

A slight alteration of the enamel surface is inevitable upon debonding of orthodontic brackets, adhesive removal, and finishing/polishing. The aim of this in vitro study was to compare two adhesives and three polishing methods by measuring enamel surface roughness using confocal laser scanning microscopy (CLSM). Brackets were bonded on 42 extracted human premolars using Transbond XT (Transbond group) or Fuji Ortho (Fuji group). After debracketing, adhesives were removed with a tungsten carbide bur, and surfaces were polished using Sof-Lex discs, a rotary brush with a prophylactic paste (Depural), or a prophylactic cup with two polishing pastes (n = 7 in each subgroup). Surface roughness (S_a_, S_ku_, S_q_, and S_z_) was measured using CLSM and compared before treatment (T_1_), after debracketing and adhesive removal (T_2_), and after polishing (T_3_). The data were statistically analyzed using the Mann–Whitney U and Kruskal–Wallis tests with Bonferroni correction. The time required for adhesive removal was measured and compared using a two-sample t-test. Surface roughness at T_2_ increased compared to T_1_, but the difference was significant only for the Fuji group (*p* < 0.01). The time required to remove Transbond XT (94.1 ± 6.8 s) was significantly higher compared to Fuji (72.1 ± 5.9 s, *p* < 0.0001). Polishing with Sof-Lex discs resulted in lower surface roughness compared to T_1_ (*p* = 0.018). Using Depural and polishing pastes showed no significant difference in surface roughness compared to T_1_, except for a significant decrease in S_a_ and S_q_ for Transbond (*p* = 0.043) and in S_ku_ for Fuji (*p* = 0.018) after polishing with Depural. In conclusion, the removal of Transbond took significantly longer, but there were fewer residues of composite resin on the enamel surface. Sof-Lex discs decreased enamel roughness, whereas enamel morphology and roughness were similar to the pre-treatment state after polishing with polishing pastes.

## 1. Introduction

To date, there is no proven method of restoring the enamel surface to its pre-treatment state after debracketing, adhesive removal, and enamel finishing/polishing. Regardless of which combination of methods is selected, enamel loss [1,2,3], change in surface roughness [4,5,6], and/or formation of cracks is inevitable [7]. Although the alteration is usually not severe [8], the damaged enamel may be more susceptible to decalcification because the superficial layer of aprismatic enamel is harder and richer in fluoride than deeper layers [9]. In addition, increased surface roughness promotes the adhesion of bacteria, which can lead to caries [10]. Therefore, it is necessary to minimize enamel damage, but at the same time, the residual adhesive should not be left on the enamel surface because that would also increase surface roughness and plaque accumulation [2,11].

The extent of enamel damage depends on the type of bur used for adhesive removal [12,13], its speed [14,15], the number of blades [16,17], or the material [5,18]. Clinically, tungsten carbide burs are commonly used [19], and they are also considered the gold standard in studies investigating enamel roughness after debonding of orthodontic brackets [4]. In contrast, the finishing and polishing of enamel is not standardized, and a wide range of tools are available. Combinations of fine rotary instruments (finishing burs, brushes, discs, polishing cups, etc.) with prophylactic or polishing pastes are often used [20,21]. For more effective polishing, a sequence of such instruments with decreasing roughness may be selected [4,5,22].

The alteration of the enamel surface may also be influenced by the adhesive used, as different adhesive types vary in adhesion mechanisms and mechanical properties. However, only a few studies examined how orthodontic adhesives affect the enamel surface [2,3,23] and the time required for their removal [24,25]. Resin composites have no chemical bond to the enamel, and their adhesion relies on micromechanical bonding ensured by acid etching and enamel adhesives [23]. Provided that no contamination of the etched surface with water or saliva occurs [26], the bond strength of enamel adhesives exceeds that of other orthodontic adhesives. Resin-modified glass ionomer cement is also commonly used because of its tolerance to humidity [27] and anti-cariogenic properties due to the release of fluoride ions. However, the chemical adhesion of glass ionomers to enamel is weak, so they are often applied in conjunction with conditioners that are able to clean and gently etch the enamel surface [28]. Without conditioning, the bond strength may be insufficient to ensure reliable adhesion of the bracket [28]. Other groups of materials, such as compomers, are used less frequently [29].

The aim of this in vitro study was to compare well-established resin composite and glass ionomer adhesives in terms of the time required for removal and the effect on enamel surface roughness after debracketing, adhesive removal, and polishing. Three widely used polishing methods were compared. The null hypotheses tested were (1) that the adhesive would affect neither the time required for removal nor enamel roughness and (2) that enamel roughness would not be affected by the polishing methods.

## 2. Materials and Methods

This in vitro study was performed with the approval of the local ethics committee (protocol number 153/21 S-IV) on 42 sound premolars extracted for orthodontic reasons in patients aged 10–16 years who underwent extraction therapy. The extracted teeth were collected with the written informed consent of the patients and their parents. After extraction, the premolars were debrided, stored in a 0.5% chloramine solution at 4–7 °C for 7 days, and then in water at 4–7 °C for a maximum of 3 months, following recommendations for the testing of adhesion of dental materials to dental tissues [30]. Only sound premolars were included in the experiment. The absence of caries, fillings, and cracks was visually assessed under a dental lamp without the use of magnification aids [18], with emphasis on the buccal (evaluated) surface. Prior to the evaluation, the surfaces were cleaned with a rotary brush, rinsed with water, and air-dried. For reliable identification of the premolars at different stages of the experiment, a number was engraved onto the root surface using a diamond bur and highlighted with a permanent marker.

Using the same technique, outlines of an area of approximately 4 × 4 mm were engraved and highlighted on the buccal surface of the premolars. This area (referred to as the orthodontic bonding area, OBA) was placed in the center of the buccal surface along the long axis of the tooth, i.e., in the area corresponding to the location of the orthodontic bracket positioning according to Mattick and Hobson [31]. Then, before bracket bonding (time T_1_), the roughness of the dried intact enamel surface was measured using a confocal laser scanning microscope (CLSM; S neox sensor, 3D Optical Profiler, Sensofar, Barcelona, Spain) placed on an anti-vibration pad, which enabled non-destructive three-dimensional evaluation [11,32,33]. A soft plastic material was used to position the evaluated surface as horizontally as possible. First, 2D images of OBA were taken at 100× and 1500× magnification using the CLSM for the qualitative evaluation of the surface. Then, a 3D map of the surface (113.34 × 94.58 µm) was recorded at five locations along the long axis of the tooth in the geometric center of each OBA at 1500× magnification. The 3D maps were used for the quantitative analysis of surface roughness using the specialized software SensoVIEW (version 1.7.0, Sensofar, Barcelona, Spain).

The specimens were then randomly divided into two equal groups using the sealed envelope method (n = 21), and a premolar bracket (Victory Series Brackets, 3M, St. Paul, MN, USA) was bonded in each OBA. Bonding was done according to the manufacturer’s instructions using two widely used adhesives [3,34]; either a light-cured resin composite (Transbond group) or a light-cured resin-modified glass ionomer cement (Fuji group), Table 1. In the Transbond group, the air-dried OBA was etched with a gel containing 35% phosphoric acid (Unitek Etching Gel, 3M, St. Paul, MN, USA) for 15 s, followed by rinsing with a water spray rinse for 15 s. After air-drying, an adhesive primer (Transbond XT Light Cure Orthodontic Adhesive Primer, 3M, St. Paul, MN, USA) was applied to OBA and light-cured with a polymerization lamp (Ortholux Luminous Curing Light, 3M, St. Paul, MN, USA) for 3 s at 1600 mW/cm^2^. A resin composite (Transbond XT Light Cure Adhesive Paste, 3M, St. Paul, MN, USA) was applied to the base of the bracket, which was then seated in OBA with moderate pressure. After removing the excess with a probe, the composite was light-cured for a total of 12 s (3 s from each side of the bracket). In the Fuji group, the air-dried OBA was conditioned with 10% polyacrylic acid (GC Ortho Conditioner, GC, Tokyo, Japan) for 20 s, followed by 20 s rinsing with water spray. OBA was gently air-dried, and a resin-modified glass ionomer cement (Fuji ORTHO LC Capsule, GC, Tokyo, Japan) was applied to the base of the bracket, which was then seated in OBA with moderate pressure. After removing the excess with a probe, the adhesive was light-cured for a total of 12 s (3 s from each side of the bracket). All specimens were incubated in water at 37 °C for 7 days [13,22,35] to ensure the proper setting of the adhesives and to simulate intraoral conditions.

Debracketing was performed with debonding pliers (Dentaurum Premium Line 004-349, Dentaurum, Ispringen, Germany) that were inserted under the occlusal and gingival wings of the bracket and pressed. This wing method was selected as it reduces the risk of enamel damage [20]. A tungsten carbide bur (123-604-30, Dentaurum, Ispringen, Germany) was used for adhesive removal at 20,000 rpm without water cooling [36], and the bur was replaced after removing the adhesive from 5 specimens. For each specimen, the time required to remove the adhesive (t_T_ for Transbond, t_F_ for Fuji) was measured with a digital stopwatch and rounded to seconds. Adhesive was considered removed when no residual material was visible on the tooth surface with the naked eye under a dental lamp [36]. Any debris was rinsed off using water spray for 10 s, and the air-dried surfaces were evaluated using CLSM (time T_2_) as described above. The tooth was positioned so that the assessed area corresponded in position and inclination to the previous measurement at time T_1_. This was verified using the 2D images at 100x magnification.

The Transbond and Fuji groups were then randomly divided into three equal subgroups (n = 7) according to the polishing method used. Sample size was estimated based on a previous study [34] and verified in our pilot study. In the Sof-Lex discs subgroup, teeth were polished using a sequence of fine and superfine polishing discs (Sof-Lex Extra Thin Fine and Superfine, 3M, St. Paul, MN, USA), each for 15 s at 20,000 rpm. Specimens from the Depural subgroup were polished for 20 s with a rotary brush (Hawe Miniature Tooth Cleaning and Polishing Brushes, Kerr, Orange, CA, USA) with a nepheline-containing prophylactic paste (Depural Neo, SpofaDental, Jičín, Czech Republic) at 3000 rpm. In the third subgroup (referred to as Polishing pastes), a prophylactic cup (Hawe Pro-Cup Hard—Dark Blue, Kerr, Orange, CA, USA) was used at 3000 rpm with two polishing pastes of descending abrasiveness; each applied for 10 s. The SuperPolish paste (Kerr, Orange, CA, USA) with Al_2_O_3_ particles was used first, followed by the CleanPolish paste (Kerr, Orange, CA, USA) with pumice powder. After rinsing and air-drying, all polished specimens were evaluated using CLSM (time T_3_) as described above.

**Figure 1:**
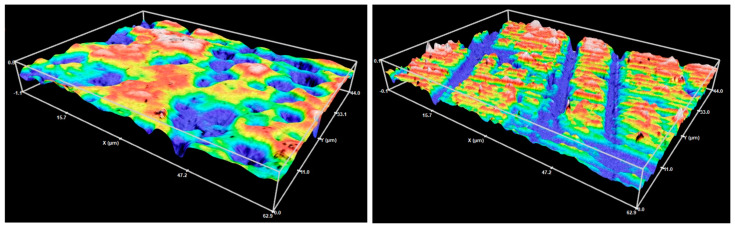
Representative 3D maps of a sound enamel surface (**left**) and enamel surface polished with Sof-Lex discs (**right**).

**Figure 2:**
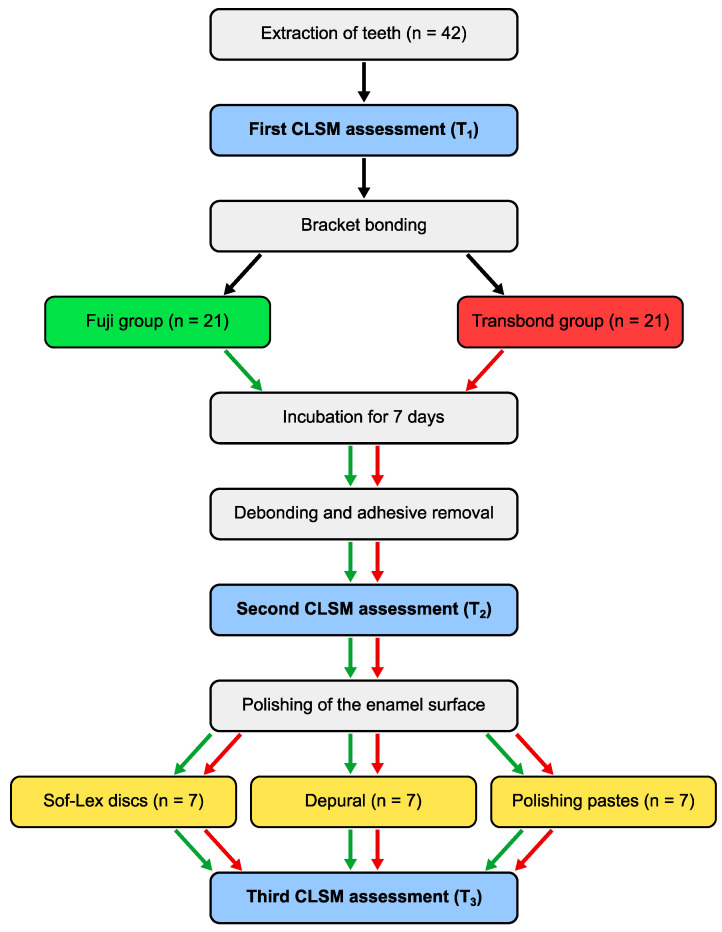
Flow chart of the experimental procedures.

The 3D maps (Figure 1) of the enamel surfaces recorded at T_1_, T_2,_ and T_3_ were analyzed using the SensoVIEW software (version 1.7.0, Sensofar, Barcelona, Spain) in accordance with ISO 25178-2:2021 [37]. With S-filter and L-filter set to 0.8 µm and 0.05 mm, respectively, the parameters S_a_, S_ku_, S_q,_ and S_z_ were calculated. All experiments and measurements were performed by the main investigator (TK). Figure 2 presents a flow chart summarizing the experimental procedures.

### 2.1. Measurement Error

To verify the repeatability of the measurements, 7 specimens were randomly selected at T_1,_ and the measurements were repeated after 4 weeks. The measurement error was expressed using Dahlberg’s formula (D) and relative Dahlberg error (RDE). In addition, the agreement between the first and second measurements was verified by the intraclass correlation coefficient (ICC), and the presence of systematic error was tested using a paired t-test. The measurement is considered sufficiently accurate if ICC exceeds 0.75 and RDE is lower than 8% [38]. The results (Table 2) showed that these criteria were met for all parameters except S_ku_ (ICC = 0.715 and RDE = 8.1%); however, the values were close to the desired criteria. The t-tests confirmed that the measurement errors were random.

### 2.2. Statistical Analysis

Medians of the five values of each parameter on each surface at each time point were calculated, as well as medians of differences in each parameter between time points (T_2_–T_1_ and T_3_–T_1_). Given the relatively small sample size in subgroups (n = 7) and the outcome of Shapiro–Wilk tests that revealed non-normal distribution in most parameters, the data were statistically analyzed using non-parametric methods. The Mann–Whitney U test was used to compare the adhesives (Transbond, Fuji), and the Kruskal–Wallis test with Bonferroni correction was used to compare the three polishing methods (Sof-Lex discs, Depural, and Polishing pastes). A comparison of surface roughness at different time points (T_1_, T_2_, T_3_) was performed using the Wilcoxon test. The data of time required to remove the adhesive (t_T_, t_F_) were normally distributed and therefore compared using a two-sample t-test. All analyses were performed using IBM SPSS Statistics for Windows (version 23.0. Armonk, IBM, NY, USA) and MedCalc (version 18.2, MedCalc Software, Ostend, Belgium) at a significance level of 0.05.

## 3. Results

Table 3 presents the comparison of surface roughness before treatment and its change after debracketing and adhesive removal. Measurements before the treatment (T_1_) showed no significant difference in any of the parameters between teeth allocated to each group (*p* > 0.3). After debracketing and adhesive removal with the tungsten carbide bur (T_2_), the values of S_a_, S_q_, and S_z_ increased, whereas S_ku_ decreased slightly. In the Transbond group, the values at T_2_ were not statistically different from those measured at T_1_ (*p* > 0.05), as opposed to the Fuji group, where values were significantly different in all parameters (*p* < 0.01). As the trend of roughness change between T_2_ and T_1_ was similar for both adhesives, their values of ΔS_a_, ΔS_ku_, ΔS_q_, and ΔS_z_ did not differ significantly (*p* > 0.1). However, the time required to remove Transbond (t_T_ = 94.1 ± 6.8 s) was significantly higher compared to Fuji (t_F_ = 72.1 ± 5.9 s), *p* < 0.0001.

Surface roughness after polishing (T_3_) with Sof-Lex discs was significantly lower than at T_1_ (*p* = 0.018), and there was also a significant decrease from T_1_ after polishing with Depural in S_a_ and S_q_ for Transbond (*p* = 0.043) and in S_ku_ for Fuji (*p* = 0.018). There was no significant difference from T_1_ in any of the parameters after using the polishing pastes with the prophylactic cup (*p* > 0.05). Table 4 summarizes changes in roughness (ΔS_a_, ΔS_ku_, ΔS_q_, or ΔS_z_) between T_3_ and T_1_. Sof-Lex discs reduced roughness significantly more than the other polishing methods (*p* < 0.05), except for ΔS_z_ values in the Transbond group polished with the polishing pastes (*p* = 0.143) and the Fuji group polished with Depural (*p* = 0.054). The change in surface roughness between T_3_ and T_1_ was similar for both adhesives when polished with Sof-Lex discs and Depural (*p* ≥ 0.18), but there was a significant difference between Transbond and Fuji in ΔS_a_, ΔS_q_, and ΔS_z_ with the polishing pastes (*p* ≤ 0.025).

**Figure 3:**
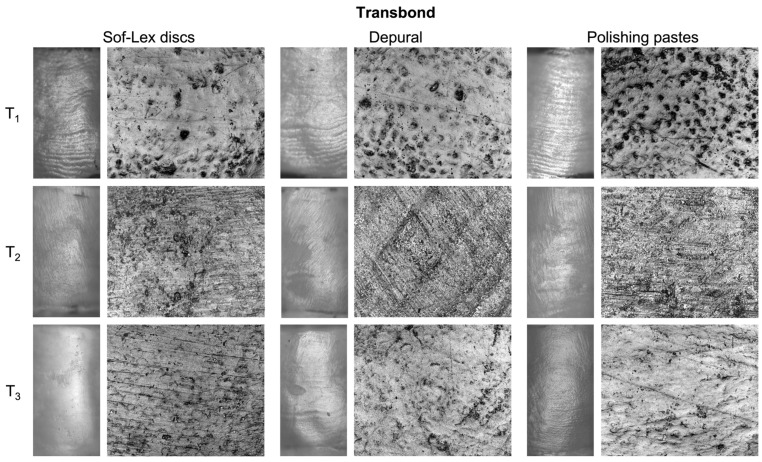
Representative images of specimens bonded with Transbond for each polishing method at each time point. In each column, images magnified 100× are presented on the left and 1500× on the right. The first row (T_1_) shows enamel surfaces before treatment—perikymata were visible at 100× magnification, while 1500× magnification revealed partial prism exposure. The second row depicts the same surfaces after debracketing and adhesive removal (T_2_)—the surfaces appeared scratched at 100× magnification, and residues of the adhesive were identified at 1500× magnification. The third row presents the surfaces after polishing (T_3_). Sof-Lex discs removed perikymata, leaving the enamel surface smooth and glossy with subtle grooves identified at 1500× magnification. Depural and polishing pastes partially preserved perikymata, and the surfaces were relatively smooth without prism exposure.

**Figure 4:**
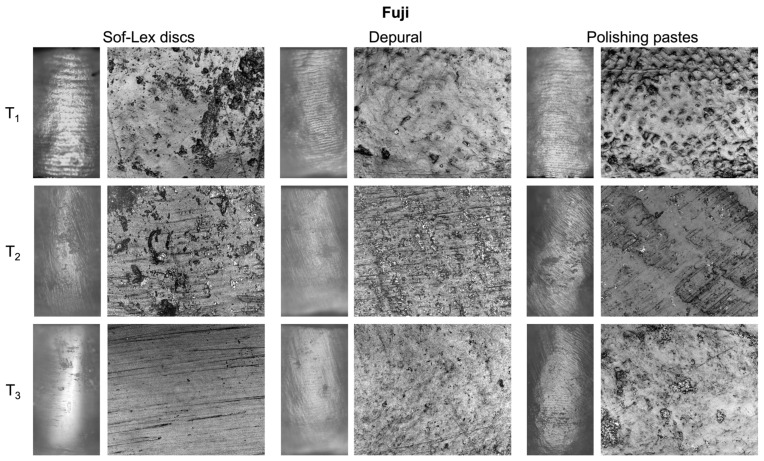
Representative images of specimens bonded with Fuji for each polishing method at each time point. For an explanation, please refer to Figure 3.

Figure 3 and Figure 4 present 2D images of representative enamel surfaces at each time point, i.e., before treatment (T_1_), after debracketing and adhesive removal with the tungsten carbide bur (T_2_), and after polishing using each of the tested methods (T_3_), for Transbond and Fuji, respectively. At T_1_, perikymata were clearly visible at 100× magnification, while the enamel microstructure with partial prism exposure was observed at 1500× magnification. At T_2_, the surface appeared scratched at 100× magnification, and residues of adhesives were found to overlie the enamel microstructure at 1500× magnification. After polishing with Sof-Lex discs, perikymata were absent, leading to a smooth and glossy appearance of the enamel surface at 100× magnification. At 1500× magnification, subtle concentric grooves corresponding to the rotation of the discs were revealed. Using Depural and the polishing pastes, perikymata were partially preserved, as shown at 100× magnification. At 1500× magnification, the enamel surface was relatively smooth, and prisms were not exposed.

## 4. Discussion

The aim of this study was to compare two types of orthodontic adhesives and three polishing methods in terms of their effect on enamel surface roughness. Prior to bonding (at T_1_), the assessment of sound enamel surfaces revealed both macroscopic and microscopic differences between individual teeth—some had more pronounced perikymata or more exposed prisms, some were smooth, and some contained subtle cracks. The quantitative analysis of the roughness parameters (S_a_, S_ku_, S_q,_ and S_z_) showed no significant difference between the teeth assigned to each group, but to prevent any influence of individual differences between teeth, the results at T_2_ and T_3_ were expressed as change in roughness parameters from baseline (T_1_).

The importance of this approach was evident in the results after debracketing and adhesive removal (T_2_). All roughness parameters in the Fuji group at T_2_ were significantly higher than at T_1_; however, the change in parameters between T_2_ and T_1_ (ΔS_a_, ΔS_ku_, ΔS_q_, ΔS_z_) was statistically similar to the Transbond group where values at T_2_ were not significantly different from T_1_. The non-significant increase in S_a_, S_q,_ and S_z_ and decrease in kurtosis (S_ku_) were explained by the 2D images—the enamel appeared lusterless. Perikymata could not be observed at 100× magnification, and parallel grooves formed by the tungsten carbide bur were visible at 1500× magnification. Comparison of results at T_2_ with other studies is not possible, as they do not evaluate surface roughness but rather the loss of enamel mass, which tends to be significantly higher when removing composites than glass ionomers [2,3].

In terms of time required for adhesive removal, it was found that removing Transbond resin composite takes significantly longer than the Fuji glass ionomer cement, which is in accordance with other studies [2,24]. On the other hand, David et al. [25] concluded that there was no significant difference in removal time between Transbond and Fuji with conditioning. Our results suggest that the time required for adhesive removal could be related to the difference in bond strength to enamel, which is higher for composites compared to glass ionomers [28,39]. This speculation is further supported by the fact that the use of a conditioner with glass ionomers was reported to increase not only the bond strength to enamel [28] but also the time required to remove the cement from the tooth surface [25].

The effect of adhesive type was also evaluated after polishing. There was no significant difference between both the groups after polishing with Sof-Lex discs and Depural, as opposed to the use of polishing pastes, which resulted in a significant difference between the adhesives in ΔS_a_, ΔS_q,_ and ΔS_z_. While the polishing pastes reduced surface roughness in the Transbond group, an increase was observed for the Fuji group. This may be caused by the higher porosity of glass ionomers compared to resin composites [40]. While the surface of the tested resin composite could be polished to a high gloss, incomplete removal of the tested glass ionomer by the polishing pastes resulted in increased surface roughness, as best observed at 1500× magnification. Since the instructions for the use of the polishing pastes state that they are designed for polishing enamel, amalgam, gold, and composite restoration, it is possible that the contained abrasives are unable to effectively remove glass particles attached to the conditioned surface. A similar result was reported by Ferreira et al. [34]. In their study, the ability of a rubber cup with a pumice stone paste to polish enamel surfaces bonded with glass ionomers was lower compared to composites. To the best of our knowledge, other similar studies are not available.

The comparison of polishing procedures was based on the extent to which the final state (T_3_) corresponded to the pre-treatment values (T_1_). With Sof-Lex discs, there was a significant decrease in S_a_, S_q,_ and S_z_, while S_ku_ increased significantly regardless of the adhesive used. The results were supported by the 2D images of the enamel—the surface was glossy, and a complete absence of perikymata was evident at 100× magnification. On the other hand, 1500× magnification revealed subtle parallel grooves corresponding to the rotation of the discs, and their presence explained the observed increase in kurtosis (S_ku_). Similar results were obtained in studies by Faria-Júnior et al. [41] and Cardoso et al. [21]—the enamel surfaces polished with Sof-Lex discs were smooth, and there was a significant decrease in R_a_ (2D roughness parameter analogous to S_a_). On the contrary, Özer et al. [42] reported a significant increase in all measured parameters after polishing with Sof-Lex discs. The conclusion of the study by Eliades et al. [22] was that Sof-Lex discs did not have a consistent effect in terms of reducing surface roughness.

Polishing using the rotary brush with Depural decreased the values of all parameters, but the decrease was significant only in S_a_ and S_q_ in the Transbond group and in S_ku_ in the Fuji group. In the 2D images, the enamel appeared naturally glossy with a hint of perikymata at 100× magnification. At 1500× magnification, the surface was relatively similar to the pre-treatment state, and enamel prisms were noticeable. This suggests that Depural applied with the rotary brush is a relatively gentle procedure that can partially restore the enamel surface. A relatively true restoration of the pre-treatment state was also achieved using the prophylactic cup with polishing pastes, as shown in the 2D images at 100× magnification. However, 1500× magnification revealed residues of the glass ionomer cement on the enamel surfaces, whereas prisms were observed in the Transbond group. Consequently, a non-significant decrease in all roughness parameters was measured in the Transbond group, while a non-significant increase was noted in the Fuji group compared to the baseline. Given the opposite trend for both adhesives, there was a significant difference between them in S_a_, S_q,_ and S_z_. 

Since numerous polishing procedures are available, comparison with other studies is complicated. Ahrari et al. [43] did not reveal any significant difference in enamel roughness prior to treatment, after adhesive removal, and after polishing with a rotary cup with a pumice paste. Cardoso et al. [21] reported that polishing with a rotary cup and a pumice paste decreased R_a_, but deeper grooves produced by a tungsten carbide bur used for adhesive removal remained on the surface. This is consistent with qualitative studies [12,44], which concluded that enamel appearance could be improved by prophylactic pastes, but major morphological surface changes created by adhesive removal tools, such as grooves and facets, cannot be entirely removed. In some studies [20,44], prophylactic pastes were used in conjunction with polishing discs, and the polished enamel surface was judged as satisfactory.

The tested polishing methods were also compared with each other, and it was found that polishing with Sof-Lex discs decreased roughness significantly more than Depural and polishing pastes, except for the ΔS_z_ values of Transbond polished with polishing pastes and Fuji polished with Depural. On the other hand, there were no significantly different changes between T_1_ and T_3_ in any roughness parameter between Depural and polishing pastes. These results are in accordance with Vidor et al. [45], who evaluated enamel roughness after Transbond removal and found no significant difference between pastes containing alumina and pumice, whereas enamel polished with Sof-Lex discs was judged as the most damaged. As for Fuji, despite no significant difference in roughness change between Depural and the polishing pastes, all roughness parameters decreased when using Depural, in contrast with the polishing pastes that increased them slightly. This supported the above speculation that the polishing pastes are unable to entirely remove the glass ionomer cement. To our knowledge, there are no other studies in which a glass ionomer adhesive was polished using polishing pastes.

The inaccuracy in targeting the same areas for roughness measurements at different time points can be seen as a limitation of this study, as well as the relatively low sample size in experimental groups, which is related to the time-consuming nature of the experiment. Furthermore, comparisons between studies are limited by the human factor because interindividual differences in bonding, adhesive removal, and polishing are inevitable. Finally, other variables have been demonstrated to have an influence on the bond strength and mechanical properties of orthodontic adhesives, such as curing light output [46] or substrate contamination [26,27]. Debracketing and adhesive removal could also be influenced by adhesive thickness [47], so the effect of these variables on roughness values should be evaluated in future studies.

## 5. Conclusions

- The removal of Transbond took significantly longer than Fuji, but there were fewer residues of Transbond on the enamel surface.
- The adhesive did not have a significant effect on the change in roughness before and after treatment except for the use of a prophylactic cup with polishing pastes that resulted in a significant lower roughness change (ΔS_a_, ΔS_q,_ and ΔS_z_) for Transbond.
- The sequential use of fine and superfine Sof-Lex discs removed perikymata, resulting in significantly lower enamel surface roughness (S_a_, S_q,_ and S_z_) compared to the situation before treatment.
- Perikymata and prisms were observed after polishing with a rotary brush with Depural. This method reduced all roughness parameters, but the differences were significant only in S_a_ and S_q_ for Transbond and S_ku_ for Fuji.
- The enamel morphology was also well restored with a prophylactic cup with the polishing pastes. However, the polishing pastes were not able to completely remove the residues of Fuji from the enamel surface.

## Figures and Tables

**Table 1:** Overview of materials used in this study.

Material (Batch Number)	Manufacturer	Material Type	Composition	Application Procedure
Unitek Etching Gel (NE06102)	3M, St. Paul, MN, USA	Etchant	Phosphoric acid (35 wt.%), water, amorphous silica	Apply to the enamel, wait for 15 s, and rinse with water for 15 s.
Transbond XT Light Cure Orthodontic Adhesive Primer (NE10102)	3M, St. Paul, MN, USA	Adhesive primer	Bis-GMA, TEGDMA, 4-(dimethylamino)-benzeneethanol	Apply to the etched enamel, light-cure for 3 s.
Transbond XT Light Cure Adhesive Paste (NE18405)	3M, St. Paul, MN, USA	Resin composite	Silane-treated quartz and silica, Bis-GMA, Bis-DMA, diphenyliodonium hexafluorophosphate, triphenylantimony	Apply to the base of the bracket, seat with moderate pressure, remove excess, and light-cure for 4 × 3 s.
GC Ortho Conditioner (2102121)	GC, Tokyo, Japan	Conditioner	10% polyacrylic acid solution	Apply to the enamel, wait for 20 s, and rinse with water for 20 s.
Fuji ORTHO LC Capsule (2105151)	GC, Tokyo, Japan	Resin-modified glass ionomer	Glass particles, HEMA, polyacrylic acid, 2-hydroxy-1,3 dimethacryloxypropane, UDMA, initiating system	Apply to the base of the bracket, seat with moderate pressure, remove excess, and light-cure for 4 × 3 s.

Abbreviations: Bis-GMA—bisphenol A-glycidyl methacrylate, TEGDMA—triethylene glycol dimethacrylate, Bis-DMA—bisphenol A dimethacrylate, HEMA—2-hydroxyethyl methacrylate, UDMA—urethane dimethacrylate.

**Table 2:** Measurement error.

Parameter	Dahlberg Error	Relative Dahlberg Error	Intraclass Correlation Coefficient	*p*-Value ^1^
S_a_	0.0077	5.9%	0.848	0.239
S_ku_	0.3028	8.1%	0.715	0.242
S_q_	0.0063	3.8%	0.933	0.189
S_z_	0.0580	5.0%	0.917	0.412

^1^ *p*-value < 0.05 would indicate systematic error.

**Table 3:** Surface roughness before treatment (T_1_) and its change after debracketing and adhesive removal (T_2_–T_1_).

		Transbond Median (min; max)	Fuji Median (min; max)	*p*-Value ^1^
T_1_	S_a_ (µm)	0.15 (0.08; 0.25)	0.17 (0.08; 0.24)	0.624
	S_ku_	3.88 (2.85; 5.28)	3.97 (3.02; 7.17)	0.333
	S_q_ (µm)	0.20 (0.10; 0.32)	0.21 (0.10; 0.32)	0.414
	S_z_ (µm)	1.51 (0.69; 2.62)	1.57 (0.75; 2.18)	0.870
T_2_-T_1_	ΔS_a_ (µm)	0.02 (−0.07; 0.27)	0.04 (−0.06; 0.17)	0.131
	ΔS_ku_	−0.55 (−1.95; 3.66)	−0.76 (−3.59; 1.01)	0.222
	ΔS_q_ (µm)	0.02 (−0.11; 0.48)	0.05 (−0.09; 0.21)	0.178
	ΔS_z_ (µm)	0.11 (−1.26; 6.00)	0.24 (−0.40; 1.16)	0.505

^1^ The *p*-value < 0.05 would indicate a significant difference between the adhesives (Transbond and Fuji).

**Table 4:** Change in surface roughness after polishing compared with pre-treatment state (T_3_–T_1_).

	Sof-Lex Discs			Depural			Polishing Pastes		
	Transbond Median (min; max)	Fuji Median (min; max)	*p*-Value	Transbond Median (min; max)	Fuji Median (min; max)	*p*-Value	Transbond Median (min; max)	Fuji Median (min; max)	*p*-Value
ΔS_a_ (µm)	−0.12 ^A^ (−0.17; −0.07)	−0.15 ^a^ (−0.18; −0.08)	0.406	−0.03 ^B^ (−0.13; 0.03)	−0.04 ^b^ (−0.09; 0.16)	0.949	−0.05 ^B^ (−0.07; 0.09)	0.06 ^b^ (−0.04; 0.18)	**0.025**
ΔS_ku_	2.60 ^A^ (1.42; 5.27)	2.16 ^a^ (0.30; 5.16)	0.180	−0.43 ^B^ (−2.15; 1.44)	−0.59 ^b^ (−4.33; −0.34)	0.406	−0.33 ^B^ (−1.82; 0.62)	0.17 ^b^ (−2.85; 1.24)	0.749
ΔS_q_ (µm)	−0.15 ^A^ (−0.21; −0.09)	−0.19 ^a^ (−0.26; −0.09)	0.180	−0.06 ^B^ (−0.17; 0.03)	−0.06 ^b^ (−0.12; 0.20)	0.749	−0.06 ^B^ (−0.10; 0.11)	0.10 ^b^ (−0.06; 0.19)	**0.025**
ΔS_z_ (µm)	−1.01 ^A^ (−1.74; −0.52)	−1.20 ^a^ (−1.58; −0.19)	0.482	−0.20 ^B^ (−0.66; 0.30)	−0.57 ^ab^ (−0.83; 0.95)	0.406	−0.59 ^AB^ (−1.09; 0.37)	0.55 ^b^ (−0.57; 1.07)	**0.009**

Within each row, different superscript letters indicate significant differences between the polishing methods (*p* < 0.05)—upper-case letters for Transbond and lower-case letters for Fuji. For example, there is a significant difference in the ΔS_q_ of Transbond between Sof-Lex discs (A) and Depural (B), but neither of them is significantly different from the polishing pastes (AB). The presented *p*-values indicate differences between adhesives for each parameter and polishing method. Significant differences (*p* < 0.05) are highlighted in bold.

## Data Availability

The data presented in this study are openly available in the Mendeley Data repository at http://dx.doi.org/10.17632/k3wybh3pz2.1, accessed on 5 January 2023.
